# Supplementary material for: Phosphonate Inhibitors of Pyruvate Dehydrogenase Perturb Homeostasis of Amino Acids and Protein Succinylation in the Brain
Source: Int J Mol Sci. 2022 Oct 29;23(21):13186. doi: 10.3390/ijms232113186 (PMC9655319; doi:10.3390/ijms232113186)
Supplement: Supplementary file 1 [file ijms-23-13186-s001.zip › ijms-1975138-supplementary.pdf]

Protein acetylation (Acet-K)

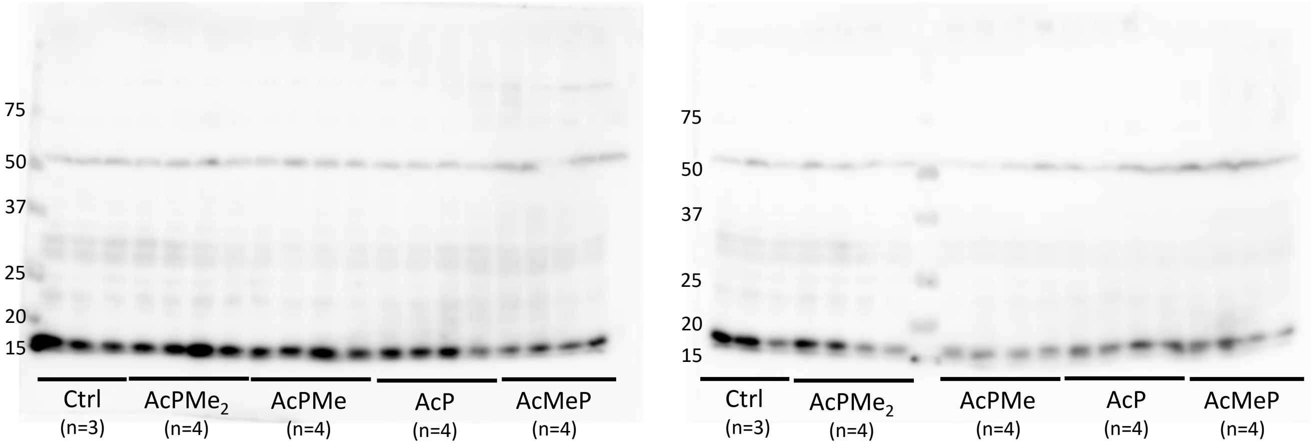

Protein siccinylation (Suc-K)

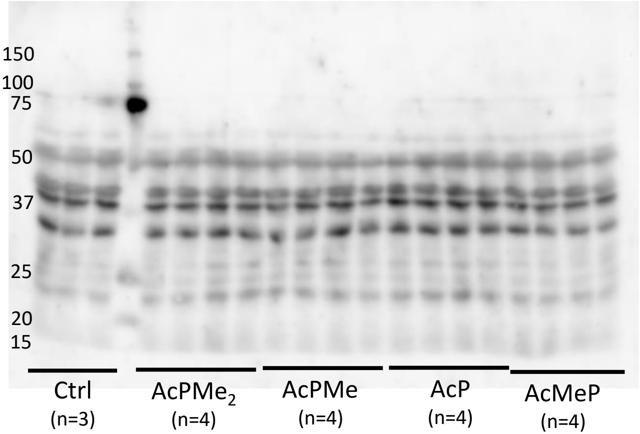

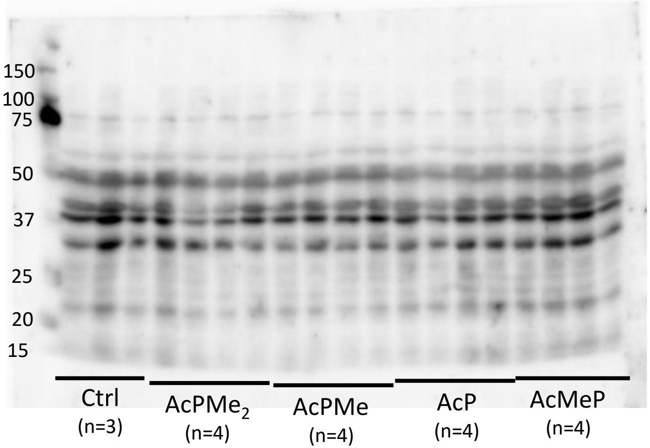

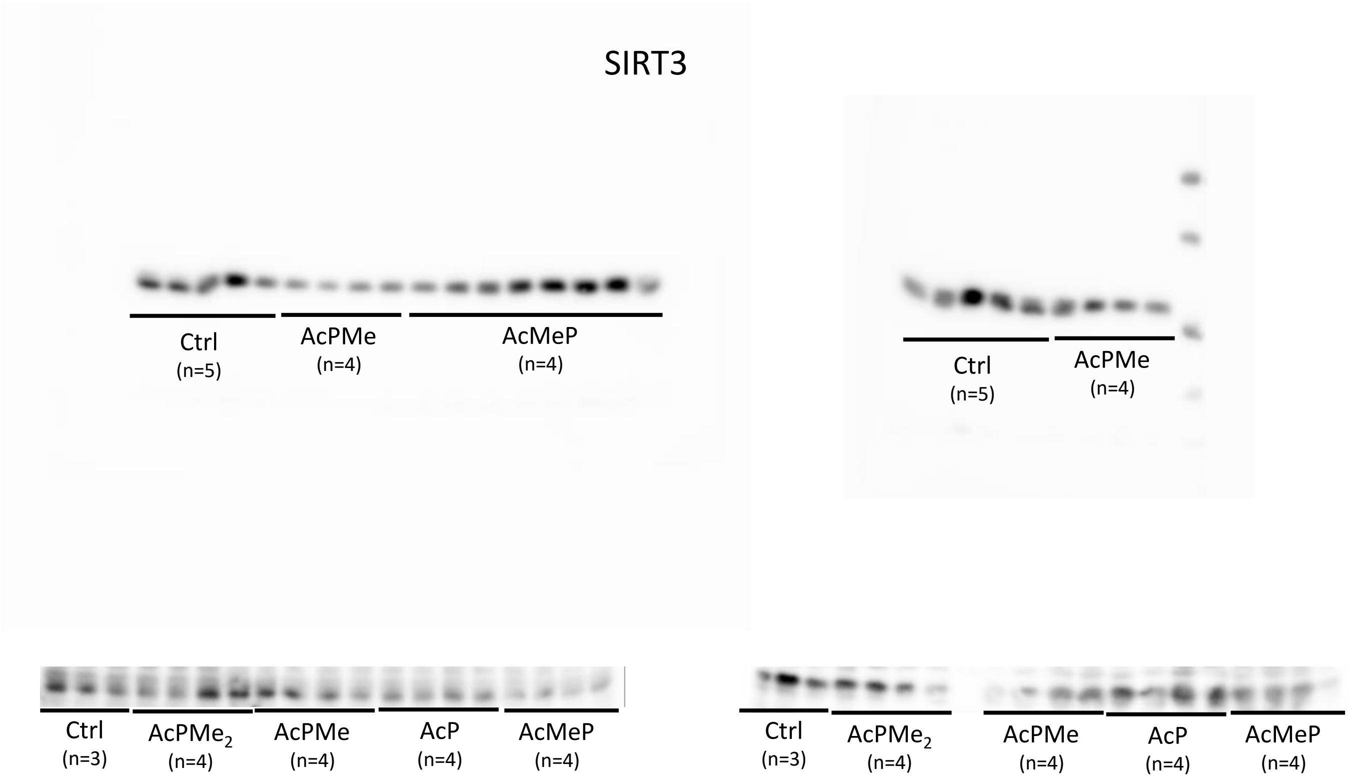
SIRT3
Ctrl (n=5)
AcPMe (n=4)
AcMeP (n=4)
Ctrl (n=5)
AcPMe (n=4)
Ctrl (n=3)
AcPMe$_2$ (n=4)
AcPMe (n=4)
AcP (n=4)
AcMeP (n=4)
Ctrl (n=3)
AcPMe$_2$ (n=4)
AcPMe (n=4)
AcP (n=4)
AcMeP (n=4)

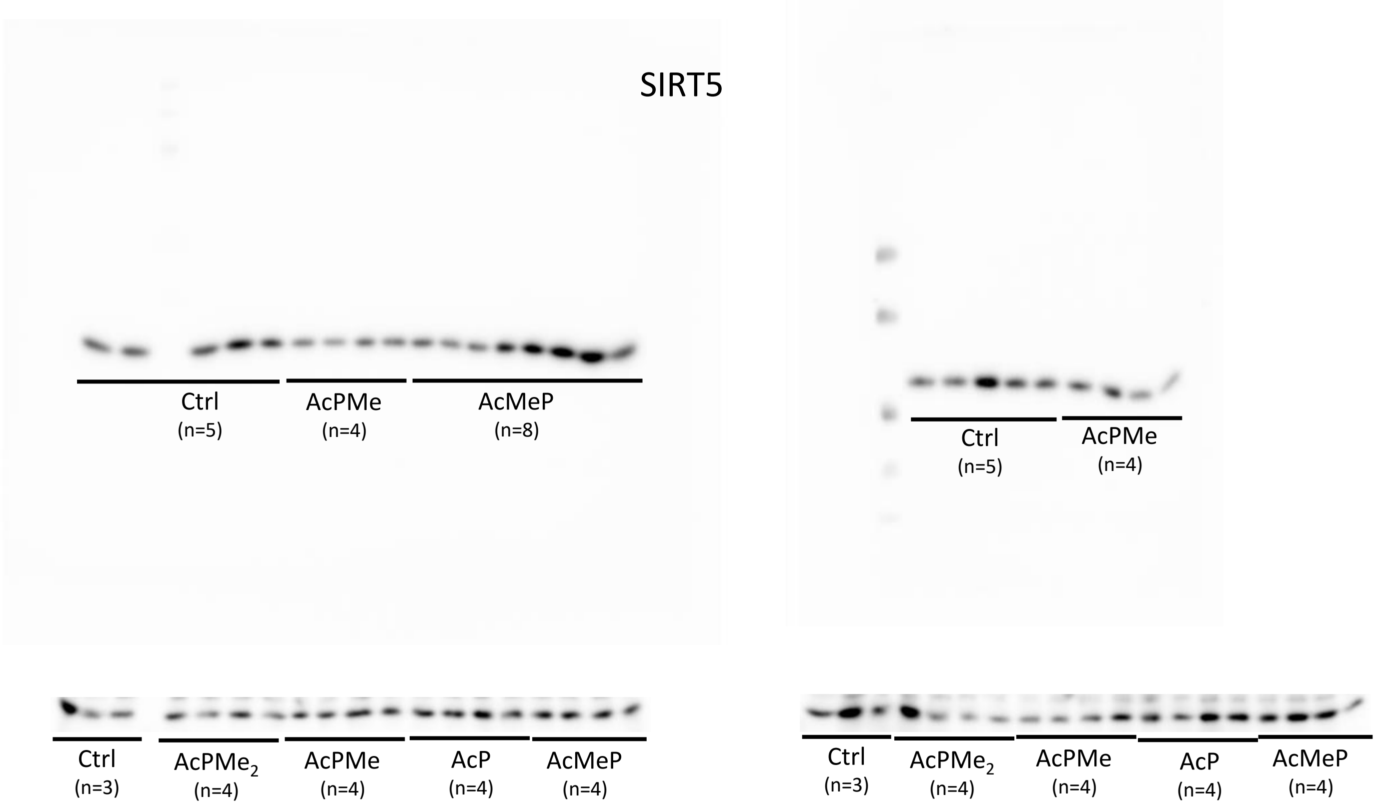
SIRT5
Ctrl
(n=5)
AcPMe
(n=4)
AcMeP
(n=8)
Ctrl
(n=5)
AcPMe
(n=4)
Ctrl
(n=3)
AcPMe$_2$
(n=4)
AcPMe
(n=4)
AcP
(n=4)
AcMeP
(n=4)
Ctrl
(n=3)
AcPMe$_2$
(n=4)
AcPMe
(n=4)
AcP
(n=4)
AcMeP
(n=4)

PDHA

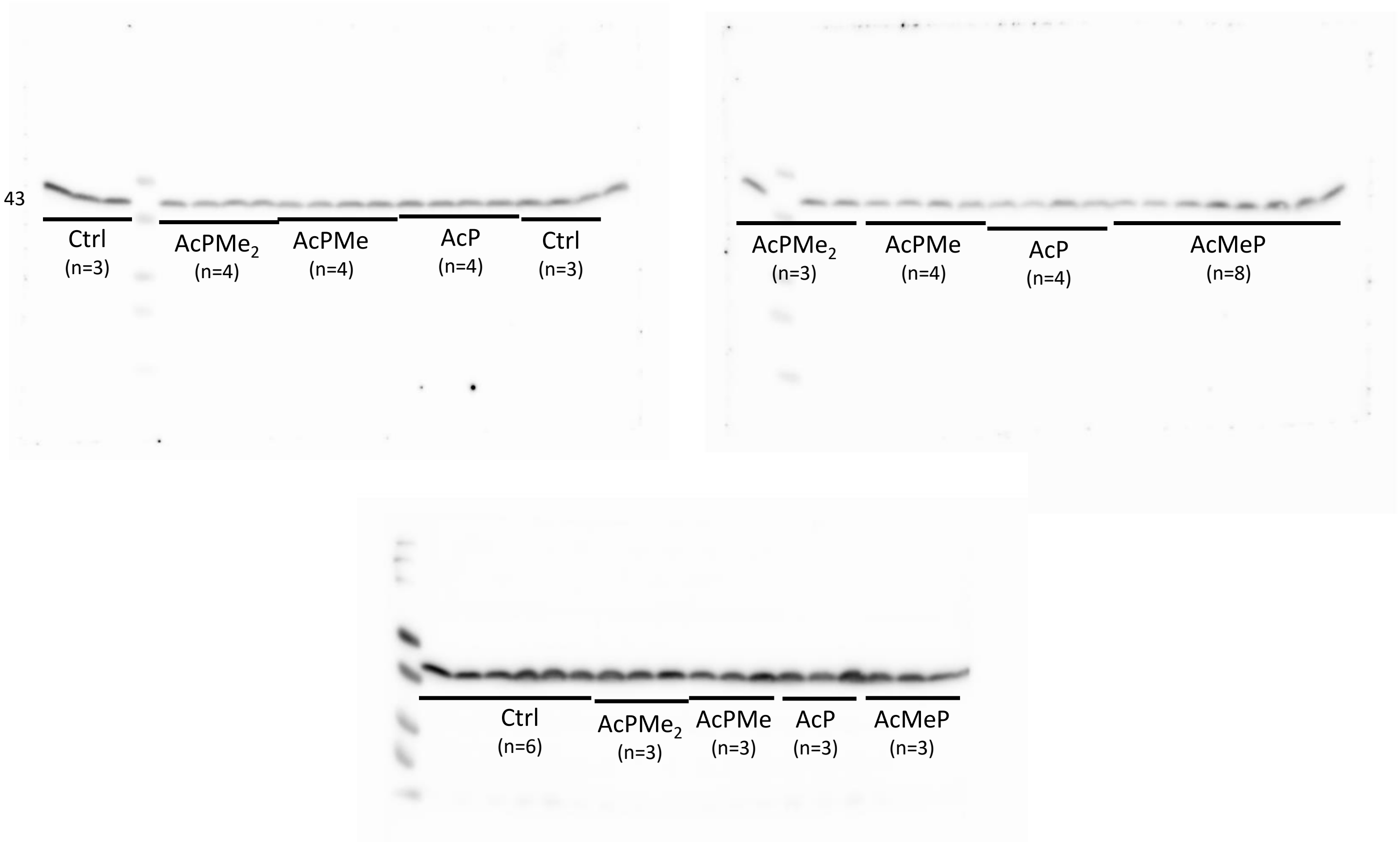
43
Ctrl (n=3)
AcPMe2 (n=4)
AcPMe (n=4)
AcP (n=4)
Ctrl (n=3)
AcPMe2 (n=3)
AcPMe (n=4)
AcP (n=4)
AcMeP (n=8)
Ctrl (n=6)
AcPMe2 (n=3)
AcPMe (n=3)
AcP (n=3)
AcMeP (n=3)

Phos-Ser293 PDHA

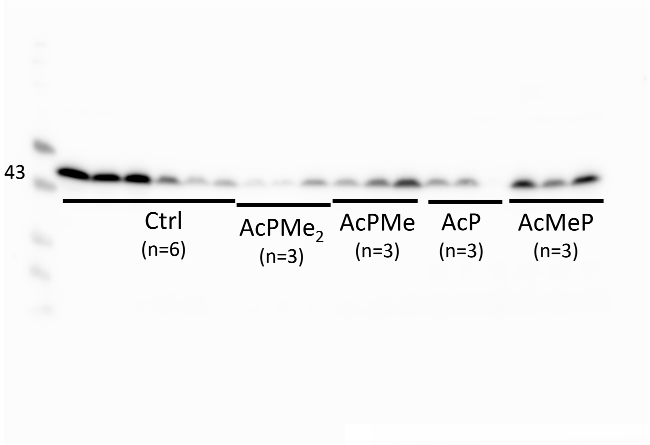

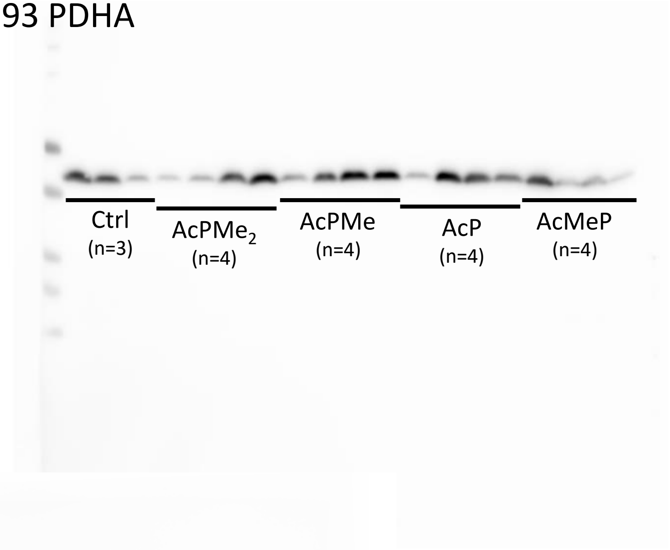

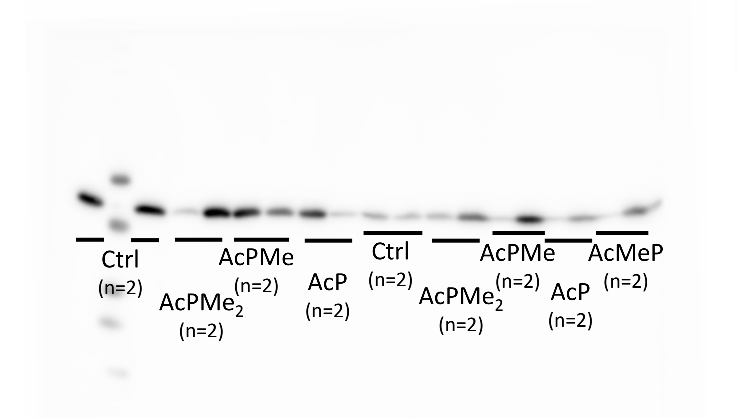

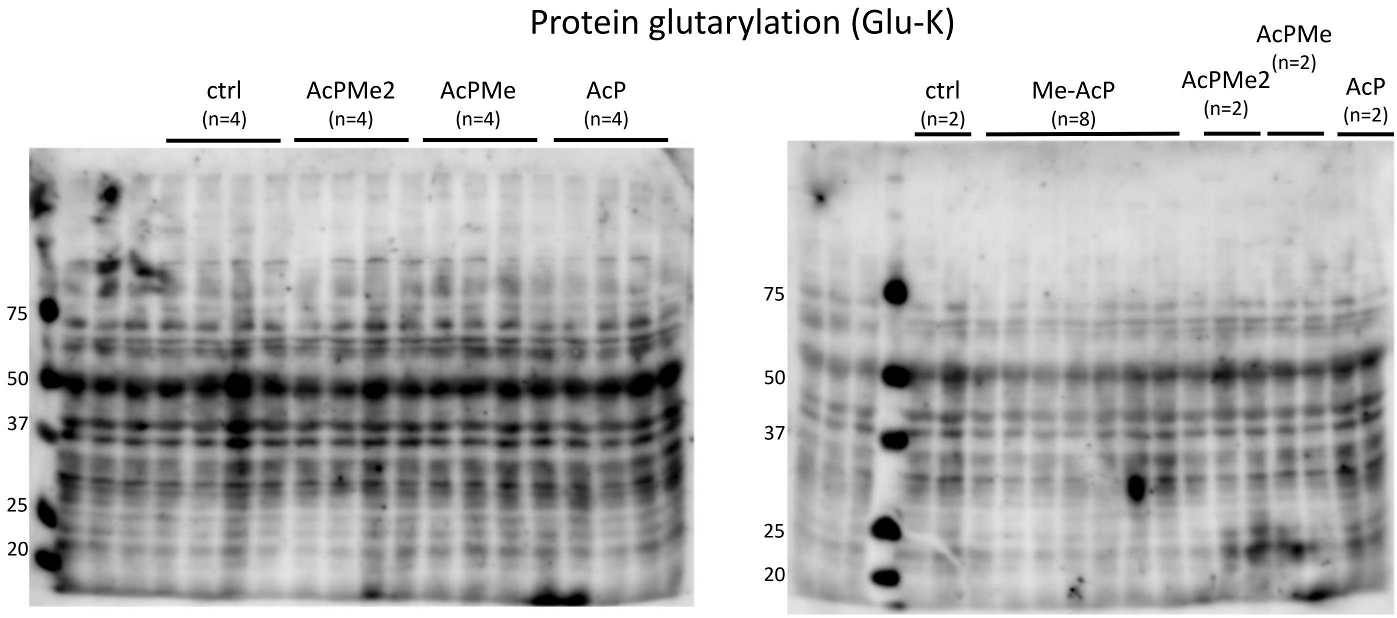

Supplementary Figure S1. Raw images of membranes after chemiluminescent detection of target proteins and modifications.
